# Ganji Formulation for Patients with Hepatocellular Carcinoma Who Have Undergone Surgery: A Multicenter, Randomized, Double-Blind, Controlled Trial

**DOI:** 10.1155/2019/9492034

**Published:** 2019-06-19

**Authors:** Jing-Hao Zhang, Chao Zheng, Xiao-Jun Zhu, Xin Zhang, Zhi-Jun Hou, Zhen-Hua Zhou, Yu-Qing Wang, Kai-Xia Wang, Zhuo Yu, Man Li, Yue-Qiu Gao, Xue-Hua Sun

**Affiliations:** ^1^Department of Hepatopathy, Shuguang Hospital, Affiliated to Shanghai University of Traditional Chinese Medicine, Shanghai 201203, China; ^2^Shanghai Traditional Chinese Medicine Clinical Center of Hepatopathy, Affiliated to Shanghai University of Traditional Chinese Medicine, Shanghai 201203, China; ^3^Laboratory of Cellular Immunity, Shanghai Key Laboratory of Traditional Chinese Medicine, Affiliated to Shanghai University of Traditional Chinese Medicine, Shanghai 201203, China

## Abstract

*Objective. *To ascertain the efficacy and safety of Ganji Formulation (GF) for patients with Hepatocellular carcinoma (HCC) who had undergone surgery.* Materials and Methods.* A total of 262 HCC patients who had undergone liver resection, local ablation, or transcatheter arterial chemoembolization (TACE) were divided randomly into the treatment group and control group. The former was treated with GF and the later with placebo, both for 6 months. The primary endpoint was overall survival (OS). Second endpoints were disease-free survival (DFS) or time to disease progression (TTP).* Results.* OS of the treatment group was significantly longer than that of the control group (*P* < 0.05). Subgroup analysis showed that, for patients who received TACE, the TTP was significantly longer in the treatment group than in the control group (P < 0.05). However, for patients who underwent liver resection or local ablation, there was no significant difference in DFS between the two groups (*P* > 0.05).* Conclusion.* GF could improve postoperative cumulative survival and prolong the TTP. This clinical trial number is registered with ChiCTR-IOR-15007349.

## 1. Introduction

Liver cancer is the fifth most common cancer and second most frequent cause of cancer-related death worldwide. Hepatocellular carcinoma (HCC) represents ~90% of primary liver cancer and constitutes a major global health problem [1]. HCC in China accounts for 55% of all HCC cases worldwide [2].

There are various treatment approaches for HCC: resection, liver transplantation, local ablative therapies, transcatheter arterial chemoembolization (TACE), particle radiotherapy, and molecularly targeted treatment [3]. Despite these options, recurrence at 5 years after curative therapies is ~70%, with a poor overall prognosis [4]. Chemotherapy and targeted therapy are associated with a low response rate, severe side effects, and expense [5]. Therefore, effective alternative and complementary approaches are required to improve the outcome for HCC patients.

Traditional Chinese medicine (TCM) has been used for centuries and has an important role in prevention of the recurrence and metastasis of cancer, attenuating toxicity, and prolonging the survival of cancer patients after surgery [6, 7]. The adjuvant therapeutic effects of some TCM herbs has been identified previously [4, 5, 8, 9]. Ganji formulation (GF) is used for HCC in TCM. However, evidence-based data regarding the efficacy and safety of GF is lacking. To address this knowledge gap, we carried out a multicenter, randomized, double-blind, placebo-controlled clinical trial to determine the therapeutic role of GF for HCC patients who had undergone surgery.

## 2. Materials and Methods

### 2.1. Ethical Approval of the Study Protocol

The present study was conducted in accordance with the ethical principles of the Declaration of Helsinki and regulation of clinical trials. The study protocol was approved by the Ethics Committee of Shuguang Hospital, Shanghai University of Traditional Chinese Medicine (2015-390-18-01; Shanghai, China). Written informed consent was obtained from all enrolled patients. The trail was registered in Chinese Clinical Trail Registry on 26th October 2015, and the clinical trial number is registered with ChiCTR-IOR-15007349.

### 2.2. GF

GF is composed of Dangshen (*Codonopsis pilosula *(Franch.) Nannf.), Baizhu (*Atractylodes macrocephala*), Baimaogen (*Imperata cylindrica Beauv.var. major (Nees) C.E.Hubb*.), Muli (*Ostreagigastnunb*), Biejia (*concha testudo graeca*), Zelan (*Aconitum gymnandrum Maxim.)*, Chenpi (*Citrus reticulata Blanco*), Maozhuacao (*Ranunculus ternatus *Thunb.), Jigucao (*Abrus cantoniensis Hance*) Baihuasheshecao (*Hedyotis diffusa Willd*) Baiying, (*Solanum lyratum Thunb*.), Banzhilian (*Scutellaria barbata D. Don*), Biliguo (*Ficus pumila Linn.*), and Tengligen (*Actinidia arguta (Sieb. & Zucc) Planch. ex Miq*.).

Concentrated granules of the 14 herbs described above were provided by Jiang Yin Tian Jiang Pharmaceuticals (Jiangsu Sheng, China) and packaged together according to the prescription of GF (lot number: 1508387). Placebo was also in the form of concentrated granules but composed of 10% GF and pharmaceutical excipient (dextrin). The latter was used in accordance with regulations on the management of pharmaceutical excipients and hygiene standards for use of food additives in China. The placebo provided by Jiang Yin Tian Jiang Pharmaceuticals shared the same package and label as GF. The appearance and taste of placebo was similar to that of herb granules.

### 2.3. Criteria for Inclusion/Exclusion

Inclusion criteria were patients aged 18–65 years; with a diagnosis of primary liver cancer (Barcelona Clinic Liver Cancer staging B or C); who had liver resection, ablation (radiofrequency ablation (RFA), microwave ablation (MWA), percutaneous ethanol injection (PEI)), or TACE 3 months previously.

Exclusion criteria were patients with metastatic liver cancer; Barcelona Clinic Liver Cancer staging D; severe complications such as refractory ascites, hepatic encephalopathy, liver failure, or portal venous embolism; other forms of liver disease; other severe primary disease or mental disorder. Also, pregnant and lactating women and patients undergoing sorafenib treatment were excluded.

### 2.4. Grouping

All patients underwent antiviral therapy, hepatoprotective therapy, diuretic treatment, or anti-infective therapy, as indicated. Patients were assigned randomly to receive GF or placebo (12 g, p.o., b.d.) for 6 months, respectively, in treatment and control groups.

### 2.5. Calculation of Sample Size and Randomization

The sample size was calculated according to the 1-year survival of 84.3% for HCC patients who had undergone surgery reported by Zhu and colleagues [10] and the assumption that the treatment group would obtain a >10% increase in survival. An alpha error of 0.05 and a study power of ~80% (beta = 0.20) were considered, and a drop-out rate of 20% was assumed. Hence, >200 patients would be required for enrollment into our study. A completely random method was employed, and enrolled patients were assigned into two groups with a proportion of 1:1, and the subjects were simply randomly assigned.

### 2.6. Follow-Up

Patients were asked to visit 3, 6, 9, and 12 months after randomization. The parameters measured were liver function; renal function; serum levels of alpha-fetoprotein (AFP); complete blood counts; urine; coagulation; Child–Pugh score; hepatitis-B virus (HBV) serology; and HBV viral load. Also, we undertook ultrasonography, computed tomography (CT), or magnetic resonance imaging (MRI) of the liver and calculated Eastern Cooperative Oncology Group performance status (ECOG-PS).

### 2.7. Efficacy Endpoints

The primary endpoint was 1-year cumulative overall survival (OS). OS was defined as the time from enrollment to death by any cause. Secondary endpoints were disease-free survival (DFS) and time to disease progression (TTP). DFS was defined as the time from randomization until any subsequent treatment for recurrent HCC diagnosed using abdominal ultrasonography, contrast-enhanced CT, or contrast-enhanced MRI. TTP was defined as the time from randomization until objective tumor progression diagnosed based on image examination and the serum level of AFP.

### 2.8. Assessment of Safety

During this trial, all patients were advised to reported changes in health and questioned about adverse events (AEs) at each follow-up point. All AEs were documented, and drug-related AEs were assessed by physicians based on physical examination and laboratory tests.

### 2.9. Statistical Analyses

The analysis was carried out according to the intention-to-treat principle. Continuous variables are the mean ± SD and were analyzed by the Student's* t*-test. Categorical variables are described by percentage. Comparison of categorical variables was conducted by Pearson's chi-square test or Fisher's exact test. The log rank test was used to compare the Kaplan–Meier survival curve between two groups. Safety analyses were done only if patients had received GF.* P* < 0.05 (two-sided) was considered significant. Statistical analyses were done using SPSS v21.0 (IBM, Armonk, NY, USA).

## 3. Results 

### 3.1. Demographic Features of the Study Population

**Figure 1:**
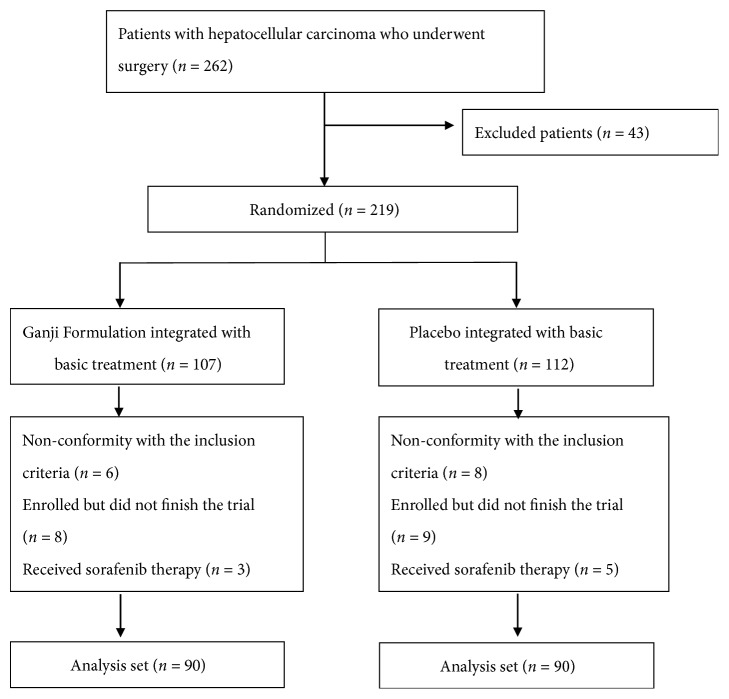
Flow diagram of the randomized clinical trial.

From September 2015 and December 2017, 262 patients were recruited from three hospitals in Shanghai. After exclusion of 43 patients, 217 eligible patients were assigned randomly to the treatment group (107) or control group (112) in a double-blinded manner. The baseline characteristics of the patients are shown in Table 1. No significant difference was observed between the groups. A flow diagram of the trial is shown in Figure 1.

### 3.2. OS

**Figure 2:**
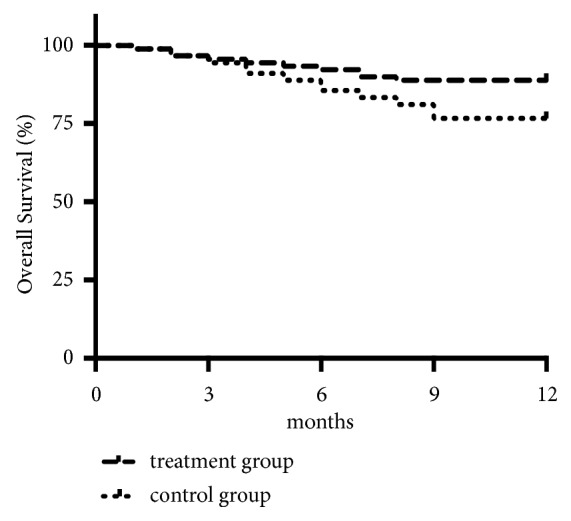
*Overall survival ratios of treatment group and control group.* Significantly better overall survival ratios were observed in treatment group than that in control group (*n*=90 per group,* P=*0.0353).

Ten patients in the treatment group and 21 people in the control group died. One-year OS was 88.9% in the treatment group* versus* 76.7% in the control group (chi-square = 4.17*, P = *0.030). Kaplan–Meier analysis demonstrated that the treatment group had significantly better cumulative OS compared with the control group (chi-square = 4.431; 95% confidence interval (CI) 0.2262–0.925;* P *= 0.0353) (Figure 2).

### 3.3. DFS and TTP

**Figure 3:**
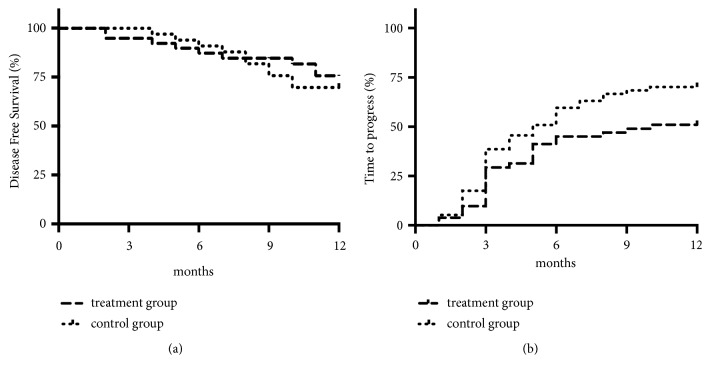
*Subgroup analysis of disease-free survival (DFS) and time to progress (TTP).* (a) DFS in patients received hepatic resection or local ablation (RFA, MWA, and PEI) between the two groups (*n*=39 in treatment group and n=33 in control group,* P*=0.488). (b) TTP in patients received TACE between the two groups (*n*=51 in treatment group and* n*=57 in control group,* P*= 0.046).

After 1 year of follow-up, for 40 patients who underwent liver resection and 32 patients who received local ablation (RFA, MWA, and PEI), 53 patients were disease-free (30 in the treatment group and 23 in the control group). No significant difference was observed in DFS between the treatment group and control group (76.9%* versus* 69.7%, chi-square=0.48;* P=*0.488) (Figure 3(a)). For 108 patients who received TACE, disease progression was observed in 26 of 51 in the treatment group and in 40 of 57 in the control group (51.0%* versus* 70.2%; chi-square = 0.442;* P = *0.041). The median TTP in the treatment group and control group was 10 months and 5.2 months, respectively. There was significantly longer TTP in the treatment group compared with the control group (chi-square = 3.983; 95%CI = 0.352–0.991;* P *= 0.046) (Figure 3(b)).

### 3.4. Safety and AEs

During follow-up, AE prevalence was similar between the two groups (Table 2). Fifty-one of the treatment group and 47 of the control group had AEs, the most being diarrhea, abdominal pain, dyspepsia, and nausea. In addition, abnormal complete blood counts (one or more than one of complete blood counts (CBC) were abnormal) were reported in both groups. All AEs were mild and tolerable.

## 4. Discussion

According to current guidelines for HCC, liver resection, liver transplantation, and local ablation are the most effective treatment methods. For patients with early-stage HCC, liver resection can result in 5-year survival of 60–80% [5], but with a prevalence of recurrence or metastasis of 40–80%. Also, most new clinically diagnosed cases are of intermediate and advanced stages, and the therapeutic options are limited to TACE, chemotherapeutic agents or radiotherapy, leading to side effects, and disappointing results [11]. Rather than focusing only on the tumor, addressing the overall physiology of the patient is a more effective approach for HCC [12].

The role of TCM as an indispensable part of the diagnosis and treatment of liver cancer has been recognized in various medical guidance documents [13]. TCM has unique advantages for the prevention and treatment of primary liver cancer, especially in the comprehensive treatment of advanced HCC. Although there is no direct or local treatment for short-term effects, a combined application can reduce the side effects of surgery, radiotherapy or chemotherapy, improve the symptoms associated with HCC, improve quality of life, and prolong survival. Previously, we showed that the basic TCM syndromes of patients with primary liver cancer who have undergone surgery are spleen deficiency, blood stasis and qi stagnation [14]. Based on previous research, combined with TCM theory for liver tumors and clinical practice, GF was established.

Here, we studied GF as an adjuvant therapy for HCC patients who had undergone surgery. All patients had Barcelona Clinic Liver Cancer staging B or C. The primary endpoint of a significant improvement in OS at 1 year with GF was observed (88.9% in the treatment group* versus* 76.7% in the control group,* P=*0.030). With regard to secondary endpoints, DFS between the treatment group and control group did not show a significant difference (*P=*0.488), and TTP in the treatment group was significantly longer compared with the control group (51.0%* versus* 70.2%,* P = *0.041). Furthermore, we observed that GF could reduce the surface antigen of the HBV (HBsAg) level in HCC patients with chronic HBV infection (data not shown).

GF is a combination of 14 herbal extracts. The active compounds of these herbal extracts can exert antitumor effects* in vivo* and* in vitro*. The underlying mechanism includes induction of mitochondria-mediated apoptosis by 2-[(2E)-3,7-dimethyl-2,6-octadienyl]-6-methyl-2,5-cyclohexadiene-1,4-dione (MDM) isolated from the aerial part of* Atractylodes macrocephala *[15] and total flavonoids from* Scutellaria barbata D. Don *[16], inducing caspase-mediated cell death by A*brus agglutinin *[17]; inhibition of proliferation of HCC cells by arrest of G2/M and G0/G1 stages of the cell cycle by nobiletin of* Citrus reticulata Blanco *[18] and total flavonoid glycosides from* Actinidia arguta* [19] and downregulation of the CDK2-E2F1 pathway by water extract of* Hedyotis Diffusa Willd* [20]; prevention of the invasion and migration of HCC cells by total saponin from root of* Actinidia valvata Dunn *[21]; enhancement of the immune system by extract of* Solanum lyratum Thunb* [22]. In addition, due to the mixture of complex compounds, multiple pathways, and targets of TCM, the effective components and specific mechanisms of actions of GF need to be clarified further.

Our trial had two main limitations. All patients were from Shanghai, and the observation period was only 1 year. Multicenter and long term follow-up clinical studies nationwide need to be carried out to further verify the clinical efficacy of GF for the patients with primary liver cancer.

## 5. Conclusions

We showed that GF combined with basic treatment could prolong OS and delay tumor progression for patients with primary liver cancer who had undergone surgery. Our study shows that GF could be adjuvant therapy for HCC.

## Figures and Tables

**Table 1:** Demographic and baseline characteristics of patients.

Characteristic	Treatment group (*n *= 90)	Control group (*n *= 90)	*P*
Age (years)^†^	56.74±8.43	55.24±10.83	0.301
Male (%)	78(86.67)	71(78.89)	0.257
Duration of illness (year)^†^	3.91±1.31	3.70±1.16	0.236
HBsAg positive (%)	76(84.44)	71(80.90)	0.060
Child–Pugh score			0.083
A	48(53.33)	37(41.11)	
B	42(46.67)	53(58.89)	
Total bilirubin (*μ*mol/L)^†^	17.51±11.24	16.07±8.91	0.387
Albumin (g/L)^†^	39.96±4.69	46.35±44.97	0.161
ALT (IU/L)^†^	51.08±42.74	53.01±37.06	0.683
AST (IU/L)^†^	47.56±42.05	52.14±39.37	0.815
*α*-fetoprotein (ng/mL)^†^	307.82±257.58	275.23±141.07	0.484
ECOG score			0.362
0	29(32.22)	27(30.0)	
1	54(60.0)	60(66.67)	
2	7(7.78)	3(3.33)	
Type of treatment			0.357
HR	22(24.44)	18(20.0)	
RFA	7(7.78)	8(8.89)	
MWA	6(6.67)	5(5.56)	
PEI	4(4.44)	2(2.22)	
TACE	51(56.67)	57(63.33)	

Notes: values in parentheses are percentages; ^†^value is the mean ± standard deviation.

ALT, alanine aminotransferase; AST, aspartate aminotransferase; ECOG, Eastern Cooperative Oncology Group; HR, hepatic resection; RFA, radiofrequency ablation; MWA, microwave ablation; PEI, percutaneous ethanol injection; TACE, transcatheter arterial chemoembolization.

**Table 2:** Adverse events during follow-up.

Adverse event	Treatment group (*n *= 90)	Control group (*n *= 90)	*P*
Overall prevalence	51(56.7)	47(52.2)	0.549
Fatigue	4(4.4)	2(2.2)	0.678
Fever	3(3.3)	2(2.2)	1
Diarrhea	7(7.8)	5(5.6)	0.55
Abdominal pain	9(10.0)	11(12.2)	0.635
Dyspepsia	10(11.1)	8(8.9)	0.619
Nausea	6(6.7)	9(10.0)	0.418
Abnormal CBC	12(13.3)	10(11.1)	0.649
Serious adverse event	0(0)	0(0)	-
Any other complains	18(20.0)	13(14.4)	0.324

Note: values in parentheses are percentages. Abnormal CBC was defined as one or more than one of complete blood counts (CBC) were abnormal. Any other complains include cough, headache, itching, dizziness, arthralgia, etc.

## Data Availability

The data used to support the findings of this study are available from the corresponding author upon request.
